# Motor Improvement-Related Regional Cerebral Blood Flow Changes in Parkinson's Disease in Response to Antiparkinsonian Drugs

**DOI:** 10.1155/2019/7503230

**Published:** 2019-03-03

**Authors:** Soutarou Taguchi, Nachi Tanabe, Jun-ichi Niwa, Manabu Doyu

**Affiliations:** Department of Neurology, Aichi Medical University, Nagakute 480-1195, Japan

## Abstract

Little is known about the relationship between regional cerebral blood flow (rCBF) change and clinical improvement in patients with Parkinson's disease (PD). Single-photon emission computed tomography (SPECT) measurement of cerebral blood flow allows evaluation of temporal changes in brain function, and using SPECT, we aimed to identify motor improvement-related rCBF changes in response to the administration of antiparkinsonian drugs. Thirty PD patients (16 without dementia; 14 with dementia) were scanned with technetium-99m labeled ethyl cysteinate dimer SPECT and were rated with the Movement Disorder Society-Unified Parkinson's Disease Rating Scale part III, both before and after a single administration of antiparkinsonian drugs. The SPECT data were processed using Statistical Parametric Mapping 2, the easy *Z*-score Imaging System, and voxel-based Stereotactic Extraction Estimation. The rCBF responses in the deep brain structures after administration of antiparkinsonian drugs tended to be larger than those in cortical areas. Among these deep brain structures, the rCBF increases in the substantia nigra (SN), lateral geniculate (LG) body, and medial geniculate (MG) body correlated with drug efficacy (*p* < 0.05, respectively). A subgroup analysis revealed that the motor improvement-related rCBF change in the MG was statistically significant, irrespective of cognitive function, but the significant changes in the LG and SN were not found in subjects with dementia. In conclusion, our SPECT study clearly exhibited drug-driven rCBF changes in PD patients, and we newly identified motor improvement-related rCBF changes in the LG and MG. These results suggest that rCBF changes in these regions could be considered as candidates for clinical indicators for objective evaluation of disease progression. Furthermore, functional studies focusing on the LG and MG, especially in relation to therapies using audio-visual stimuli, may bring some new clues to explain the pathophysiology of PD.

## 1. Introduction

Parkinson's disease (PD) is a chronic progressive neurological disease manifesting with motor symptoms of resting tremor, rigidity, bradykinesia, and postural instability, as well as nonmotor symptoms such as constipation, orthostatic hypotension, and hallucination [1]. As the main pathophysiology of PD is degeneration and loss of dopaminergic neurons, dopaminergic agents including L-DOPA are commonly used for its pharmacological treatment. The effects of antiparkinsonian drugs change in a temporal manner, in accordance with their pharmacokinetics.

Nuclear imaging studies including single-photon emission computed tomography (SPECT) and positron emission tomography are widely used to assess the spreading of parkinsonian brain lesions from a functional perspective. Although SPECT has recently been used to evaluate dopamine transporters, it is more widely used to measure cerebral blood flow (CBF). CBF SPECT can facilitate the visualization of brain activity and allow the quantitative evaluation of temporal changes in brain function. Therefore, CBF SPECT presents the possibility of obtaining detailed and objective evaluations of the functional changes occurring in the parkinsonian brain, immediately after a single administration of antiparkinsonian drugs. However, little is known about the relationship between improvement in clinical features and the CBF changes occurring immediately after a single administration of antiparkinsonian drugs to PD patients under long-term antiparkinsonian treatment. Previous studies have been subject to various limitations, including a small number of subjects, small numbers of volumes of interest (VOIs), poor spatial resolution, subjective selection of VOIs, long withholding of medication, unusual drug dosage, less reliable and valid rating scales for clinical features, and a heavy physical burden on the patients [2–12]. Previously, we used conventional SPECT methods to show that antiparkinsonian drugs significantly increased regional CBF (rCBF) in the lenticular nucleus of PD patients, while decreasing it in the frontal cortex [13]. Unfortunately, in this previous study, the SPECT acquisitions were made on separate days, and it was not possible to directly observe the temporal CBF changes occurring immediately after a single administration of antiparkinsonian drugs.

In this study, to identify motor improvement-related rCBF changes in response to antiparkinsonian drugs, we used a recent more comprehensive observer-independent neuroimaging technique to investigate the motor and rCBF responses occurring immediately after a single administration of antiparkinsonian drugs [14–16]. This also involved SPECT acquisitions performed before and after drug administration, on the same day. Furthermore, we also analyzed how dementia could affect these rCBF responses [17].

## 2. Materials and Methods

### 2.1. Subjects

Thirty-four PD patients under long-term antiparkinsonian treatment who were registered at the Aichi Medical University Hospital were enrolled in this study. The diagnosis of PD was made according to the Movement Disorder Society (MDS) clinical diagnostic criteria for PD (clinically established or probable PD) [18]. Four patients in whom severe brain atrophy or intracranial pathology (e.g., stroke) were observed on magnetic resonance imaging (MRI) were excluded from the analysis because their atrophy or lesions may have influenced the SPECT count (i.e., a partial volume effect). Therefore, 30 PD patients were included in the study, with these being summarized in Table 1. Of the thirty cases, 16 were PD patients without dementia (PD-ND subgroup) and 14 were PD patients with dementia (PD-D subgroup) according to the MDS diagnostic procedure [19]. The characteristics of the two subgroups are also summarized in Table 1. The prevalence of hallucinations was significantly higher in the PD-D subgroup (86%) than in the PD-ND subgroup (13%, *p* < 0.05; Table 1).

### 2.2. SPECT Protocol

CBF was measured using technetium-99m labeled ethyl cysteinate dimer (^99m^Tc-ECD; FUJIFILM RI Pharma Co. Ltd., Tokyo, Japan) SPECT with a dual-head rotating gamma camera (Infinia Elite™ detector, General Electric Co., New York, USA) and a low-energy high-resolution collimator. The scanning parameters included photo peak, 140 keV ±10%; acquisition method and time, 6° × 120 frames per head over 360° × 2, and 10 s/frame using step-and-shoot; acquisition matrix size, 128 × 128; magnification × 1.5; and pixel size, 2.8 × 2.8 mm. Every patient was scanned twice on the same day, before (off-stage) and after (on-stage) oral administration of the normal daily first morning dose of antiparkinsonian drugs, with the antiparkinsonian drugs being withheld for at least 10 hours prior to the off-stage scanning. Therefore, all the off-stage SPECT was acquired in the morning. The two SPECT acquisitions were made around 3.5 hours apart, with ^99m^Tc-ECD tracer (off-stage, approximately 480 MBq; on-stage, approximately 280 MBq) being injected via the right cubital vein. With consideration of the ^99m^Tc-ECD decay, the acquisition times were 15 min in the off-stage and 30 min in the on-stage, to equalize the counts between them. In each patient, the daily first morning dose of antiparkinsonian drugs was adjusted to facilitate immediate transitioning of the patient from the off-stage to on-stage.

### 2.3. Clinical Evaluation

The patient's motor signs and symptoms were assessed with the MDS-Unified Parkinson's Disease Rating Scale (MDS-UPDRS) [20]. The motor performances of the patients were rated prior to each scan by a single neurologist who passed the MDS-UPDRS training program and certification exercise. To confirm the objectivity and accuracy of the clinical evaluations, each examination was checked by the other neurologists. After the single oral administration of antiparkinsonian drugs, motor improvement was quantitatively assessed according to the “*Motor Improvement Index*,” which was defined as: ([MDS-UPDRS part III in the off-stage] – [MDS-UPDRS part III in the on-stage])/[MDS-UPDRS part III in the off-stage].

### 2.4. SPECT Data Analysis

The SPECT data were processed using Statistical Parametric Mapping 2 (SPM2) [14], the easy *Z*-score Imaging System (eZIS®; FUJIFILM RI Pharma Co. Ltd., Tokyo, Japan) [15], and voxel-based Stereotactic Extraction Estimation (vbSEE®; FUJIFILM RI Pharma Co. Ltd.) [16]. These software tools made it possible to detect detailed, comprehensive, and highly sensitive rCBF changes in an observer-independent manner. Using SPM2 software, individual SPECT acquisitions were spatially normalized to Montreal Neurological Institute coordinates with a voxel size of 2.0 × 2.0 × 2.0 mm and then smoothed with a Gaussian filter. Using eZIS® software, the spatially normalized and smoothed data of the regional ^99m^Tc-ECD uptake of every patient were normalized to the global mean of the brain and then statistically compared with a normal database, allowing the *Z*-score for each voxel to be calculated. The *Z*-score was defined as ([the average value of the normal database] − [the value of the individual patient])/[the standard deviation (SD) of the normal database]. These *Z*-score maps of the brain were segmented into VOIs in accordance with Talairach atlas space using vbSEE® software [21, 22]. The “Decrease-Extent,” defined as the percentage of voxels with a relatively low SPECT count (*Z*-score > 2.0) within a VOI, and the “Increase-Extent,” defined as the percentage of voxels with a relatively high SPECT count (*Z*-score > 2.0), were quantified for each VOI. Finally, the rCBF change in each VOI after oral administration of the antiparkinsonian drugs was analyzed as the “*rCBF change index*” (% points), with this being defined as [Decrease-Extent (%) in off-stage] − [Decrease-Extent (%) in on-stage] or [Increase-Extent (%) in on-stage] − [Increase-Extent (%) in off-stage]. A mean *rCBF change index* > 0 signifies an rCBF increase under antiparkinsonian drugs, and a value < 0 signifies an rCBF decrease.

### 2.5. Statistical Analysis

Statistical analysis of the data was performed using JMP version 5.0.1a (SAS Institute Inc., North Carolina, USA). Pearson's chi-squared test and the nonparametric Mann–Whitney *U* test were used for between subgroup comparisons of categorical data (gender ratio and the prevalence of motor complications, hallucination, depression, anxiety, and orthostatic hypotension), and age (years), disease duration (years), MDS-UPDRS score (points), dosage of antiparkinsonian drugs (LED, levodopa equivalent dose[23], mg), and Hoehn and Yahr stage [24]. Spearman's rank correlation coefficient was used to test for correlations between SPECT changes (*rCBF change index* (% points)) and motor improvement (*Motor Improvement Index*), and between dosage of antiparkinsonian drugs (LED, mg) and motor improvement (*Motor Improvement Index*). The Wilcoxon signed-rank test was used to evaluate motor performance (MDS-UPDRS part III score (points)) in the off-stage versus in the on-stage, and rCBF (Extent (%)) in the off-stage versus in the on-stage. The level of significance was set at *p* < 0.05.

### 2.6. Ethical Statement

This study was approved by the ethical review board of Aichi Medical University (no. 14-163) and conformed with the Declaration of Helsinki. All participants provided written informed consent.

## 3. Results

**Figure 1:**
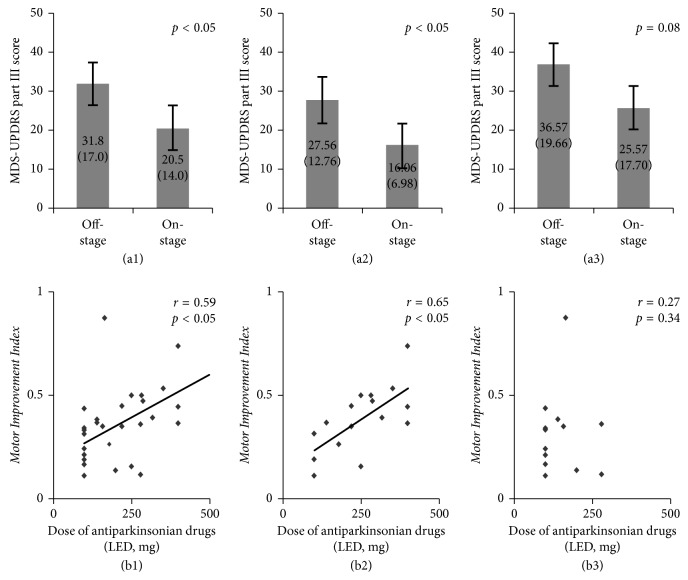
Motor performance improved in each of the 30 patients (*p* < 0.05) (a1). Improvements in the individual motor performances in the 30 PD patients positively correlated with individual doses of antiparkinsonian drugs (*r* = 0.59, *p* < 0.05) (b1). These statistically significant improvements were also seen in the PD-ND subgroup (*p* < 0.05 (a2); *r* = 0.65, *p* < 0.05 (b2)) but were not present in the PD-D subgroup (*p*=0.08 (a3); *r* = 0.27, *p*=0.34 (b3)). LED, levodopa equivalent dose.

Motor performance improved in each of the 30 patients after administration of their normal daily first morning dose of antiparkinsonian drugs (MDS-UPDRS part III score: in the off-stage, mean 31.8 [SD 17.0]; in the on-stage, mean 20.5 [SD 14.0]; see Figure 1(a1)). Improvements in the individual motor performances of the 30 PD patients were positively correlated with individual doses of antiparkinsonian drugs (*r* = 0.59, see Figure 1(b1)). These significant improvements were also seen in the PD-ND subgroup (MDS-UPDRS part III: in the off-stage, mean 27.56 [SD 12.76]; in the on-stage, mean 16.06 [SD 6.98], see Figure 1(a2); *r* = 0.65, B2) but were not present in the PD-D subgroup (see Figures 1(a3) and 1(b3)).

**Figure 2:**
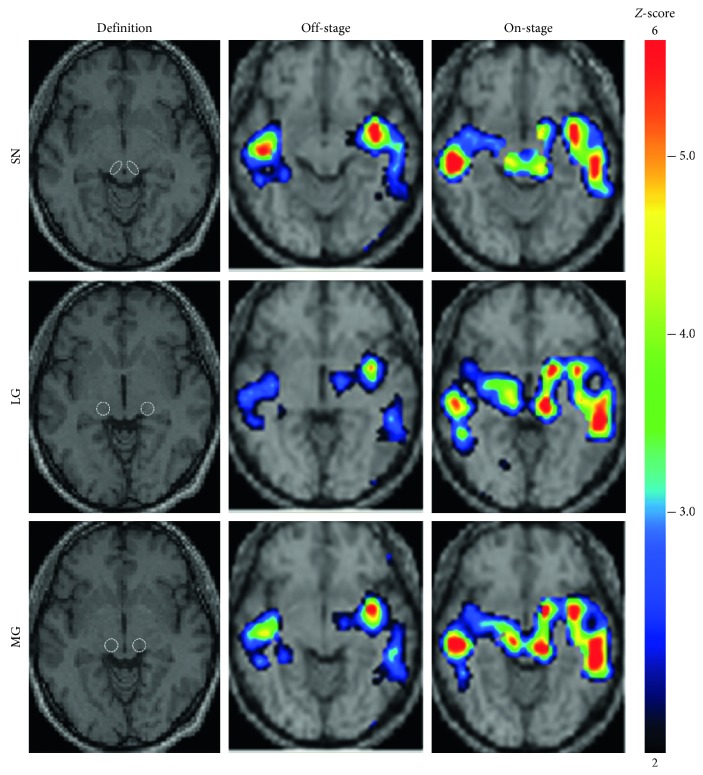
*Z*-score maps from a typical patient. An 81-year-old female (with M/C, depression, and anxiety; without dementia, hallucination, or orthostatic hypotension; Movement Disorder Society's Unified Parkinson's Disease Rating Scale part III: 49 points in the off-stage and 27 points in the on-stage; levodopa equivalent dose 420 mg/day; Hoehn–Yahr stage 4) showed regional cerebral blood flow increase in the SN, LG, and MG in response to antiparkinsonian drugs. LG, lateral geniculate body; MG, medial geniculate body; SN, substantia nigra.

For the entire group of 30 PD patients, the regions (according to the VOIs based on the Talairach atlas space) where CBF showed significant change after administration of antiparkinsonian drugs are summarized in Table 2. Antiparkinsonian drugs significantly increased rCBF in the putamen (Put), external segment of the globus pallidus (GPe), internal segment of the globus pallidus (GPi), and the substantia nigra (SN) in the basal ganglia; the lateral geniculate (LG) body, medial geniculate (MG) body, pulvinar (Pul), ventral anterior nucleus (VA), and ventral posterior nucleus (VP) in the thalamus; and the insula (Ins), primary visual cortex (PVC), red nucleus (RN), and somatosensory association cortex (SSAC). In contrast, within the frontal cortex, there was a slight increase in rCBF in the dorsolateral prefrontal cortex (DLPFC) but decreased rCBF in the anterior cingulate cortex (ACC), inferior frontal gyrus (IFG), and orbitofrontal cortex (OFC). Among those regions where CBF changed in association with antiparkinsonian drugs, correlations between motor improvement and rCBF increase were shown in the SN (*r* = 0.37), LG (*r* = 0.60), and MG (*r* = 0.54; see Table 2). The *Z*-score maps of a typical patient are presented in Figure 2.

Subgroup analysis was performed to examine how the dementia affected the rCBF increase in the SN, LG, and MG, and its correlation with motor improvement. The magnitude of rCBF increase in the SN in the PD-ND subgroup was larger than in the PD-D subgroup. Significant correlations between motor improvement and rCBF increase in the SN disappeared in both subgroups (see (a) in Table 3). In the LG, as in the SN, the magnitude of rCBF increase in the PD-ND subgroup was larger than in the whole group of 30 PD patients, whereas in the PD-D subgroup, the magnitude of rCBF increase showed a trend towards being smaller, although it did not reach statistical significance, and there was no significant correlation with motor improvement (see (b) in Table 3). In contrast to these two regions, the rCBF increase in the MG and its correlation with motor improvement remained statistically significant, irrespective of cognitive function (see (c) in Table 3).

## 4. Discussion

In the present study, we analyzed motor improvement and rCBF change immediately after a single administration of antiparkinsonian drugs, evaluating correlations between them and examining how they were affected by dementia.

In the 30 PD patients, the MDS-UPDRS part III score significantly improved by 11.3 points after administration of antiparkinsonian drugs, with this improvement significantly and positively correlating with the dose of the drugs. To evaluate SPECT rCBF change in response to these antiparkinsonian drugs, the regions where a CBF response occurred in relation to a single administration of the drugs were screened according to VOIs based on Talairach atlas space. The results showed that antiparkinsonian drugs significantly increased rCBF in VOIs belonging to the basal ganglia and thalamus but decreased rCBF in VOIs belonging to the frontal cortex. These findings are consistent not only with our previous report [13] but also with a number of previous studies by other authors [4–7, 10–12]. Furthermore, a meta-analysis of functional MRI studies also reported regional abnormalities in brain function in the OFC and SSAC in off-stage PD patients [25]. The rCBF responses in VOIs belonging to the deep brain structures tended to be larger than those in cortical VOIs. Direct contact between dopaminergic nerve terminals and microvasculature is proposed as one of the mechanisms for the cerebrovascular response occurring with antiparkinsonian drugs. Therefore, the difference in the rCBF response between cortical areas and deep brain structures could be related to the density of dopaminergic innervation to the microvasculature, or the degree of neurodegeneration [26]. Moreover, our present study newly identified the LG and MG as regions where CBF significantly increased with administration of antiparkinsonian drugs. Interestingly, not only was an rCBF increase in the SN significantly correlated with motor improvement but so were also rCBF increases in the LG and MG. This suggests that rCBF changes in these three regions reflect objective and quantitative parkinsonian motor improvement caused by antiparkinsonian drugs.

The SN belongs to the corticobasal ganglia motor loop involved in locomotion, which is obstructed in PD [27]. Therefore, it seems reasonable to assume that this region functionally responded to antiparkinsonian drugs in the PD patients. However, the LG, which has neural connections with the PVC, is closely associated with the limbic system and frontal cortex via the ventral pathway [28], and the MG has neural connections with the spine [29] and striatum [30], and it is suspected that these two regions deal with functions other than visual and auditory ones. Although no report has clearly explained the association of the LG or MG with the extrapyramidal function, it is well known that therapies involving audio-visual stimuli (e.g., visual and auditory cues) can dramatically improve performance in PD patients [31]. Therefore, further research to explain our findings is required, and future studies focusing on the involvement of the LG or MG in various parkinsonian brain functions may bring some new clues towards explaining the pathophysiology of PD.

We also examined how dementia affected the motor improvement and rCBF change occurring with antiparkinsonian drugs. The statistically significant motor improvement seen in the PD-ND patients was not present in the PD-D patients. This may be due to a loss of statistical power because of the small numbers in the PD-D subgroup (type II error). However, other possible factors include insufficient dosage of antiparkinsonian drugs, older age, longer disease duration, and greater severity of motor dysfunction in comparison with the PD-ND subgroup, although none of these factors showed significance (see Table 1). The dosage of antiparkinsonian drugs administered in the present study (445.1 mg LED) is almost the same as that shown in a large-scale survey of Japanese PD patients (421 mg LED) [32], and the variation in the MDS-UPDRS part III score was 11.0 points, which was almost equal to the PD-ND subgroup (11.5 points), which means that the PD-D patients were sufficiently in the “on-state.” Therefore, the influence of these aforementioned factors is assumed to be minor. Moreover, in the PD-D subgroup, the significant correlation between motor improvement and dose of antiparkinsonian drugs disappeared, with the data showing substantial scatter (Figure 1(b3), *r* = 0.27). We therefore consider that the nonsignificant correlation was not merely due to the small number of patients and that even if the patient number was increased, the correlation coefficient would still not reach significance in the PD-D subgroup. Therefore, we believe that dementia might affect dose-dependent motor improvement, at least to the extent of the loss of a significant correlation between dose and motor improvement; in other words, dementia could interfere with the motor improvement attained by the use of antiparkinsonian drugs in PD patients.

It is important to elucidate how dementia could interfere with the motor improvement obtained from antiparkinsonian drugs and how this is reflected in parkinsonian brain function. In the present subanalysis based on dementia, we found that although the rCBF increase in the SN was significant regardless of cognitive function, the magnitude of the rCBF increase was larger in the PD-ND patients than in the PD-D patients, and significant correlations between motor improvement and rCBF increase disappeared in both the subgroups. In the LG, the significance of both the rCBF increase and its correlation with motor improvement disappeared in the PD-D subgroup. Although the reason for this is unknown, when the fact that 86% of the PD-D patients suffered hallucinations is considered in association with the fact that the LG is a part of the visual pathway, hallucination might have affected rCBF change in the LG [33, 34]. In the MG, rCBF showed not only a significant increase immediately after a single administration of antiparkinsonian drugs but also a significant correlation with motor improvement in both PD-ND and PD-D subgroups. These results indicate that among the three regions, the motor improvement-related rCBF changes in the MG were the most stable, irrespective of the patient's cognitive function. The relevance of rCBF changes in the MG of the PD brain is of course vague at this moment, but this finding may exist in cases without parkinsonian symptoms, such as patients in the prodromal stage. Furthermore, we may be able to use this finding as an objective marker to follow the clinical progression of PD patients under antiparkinsonian drug treatment. Further studies with sufficient patient numbers to allow multivariate analysis are required.

## 5. Conclusions

Our SPECT study clearly demonstrated drug-driven rCBF changes in PD patients, and we newly identified motor improvement-related rCBF changes in the LG and MG. These results suggest that rCBF changes in these regions could be considered as candidate clinical indicators for objectively evaluating the clinical progression of PD. Furthermore, functional studies focusing on the LG and MG, especially in relation to therapies using audio-visual stimuli, may present some new clues to explain the pathophysiology of PD.

## Figures and Tables

**Table 1:** Characteristics of the 30 PD patients and the subgroups according to the presence of dementia.

	Total 30 PD patients	PD-ND subgroup	PD-D subgroup
*n* (gender, men: women)	30 (19 : 11)	16 (9 : 7)	14 (10 : 4)
Age when examined (years)	73.5 (SD 8.2)	71.3 (SD 8.5)	76.1 (SD 6.9)
Disease duration (years)	6.4 (SD 6.3)	4.6 (SD 3.1)	8.5 (SD 8.1)
Mean MDS-UPDRS total score in the off-stage (points)	65.4 (SD 31.9)	54.6 (SD 22.3)	78.1 (SD 36.2)
Mean MDS-UPDRS part III score in the off-stage (points)	31.8 (SD 17.0)	27.6 (SD 12.8)	36.6 (SD 19.7)
Daily dosage of antiparkinsonian drugs (LED, mg)	445.1 (SD 241.6)	469.5 (SD 252.3)	417.1 (SD 225.4)
Hoehn and Yahr stage in the off-stage (points)	2.6 (SD 0.9)	2.4 (SD 0.8)	2.7 (SD 0.9)
MMSE, total score (points)	25.2 (SD 3.8)	28.3 (SD 1.4)	21.6 (SD 2.3)^†^
M/C (%)	50	50	50
Hallucination (%)	47	13	86^†^
Depression and anxiety (%)	67	69	64
Orthostatic hypotension (%)	63	69	57

^†^Significant difference between the two subgroups (*p* < 0.05). LED, levodopa equivalent dose; M/C, motor complications; MDS-UPDRS, Movement Disorder Society's Unified Parkinson's Disease Rating Scale; MMSE, Mini-Mental State Examination; PD, Parkinson's disease, PD-D, PD with dementia; PD-ND, PD without dementia; SD, standard deviation.

**Table 2:** rCBF change in response to antiparkinsonian drugs and its correlation with motor improvement in the 30 PD patients.

VOI	Mean *rCBF change index* (% points)^†^	*r*
*Basal ganglia*		
Put	18.65 (SD 20.16)	0.28
Gpe	16.97 (SD 24.38)	0.16
Gpi	8.38 (SD 22.81)	0.04
SN	31.10 (SD 32.36)	0.37^†^
*Frontal cortex*		
ACC	−3.94 (SD 8.15)	0.45
DLPFC	2.16 (SD 3.15)	0.19
IFG	−4.65 (SD 11.54)	0.18
OFC	−6.30 (SD 10.11)	0.67
*Thalamus*		
LG	17.22 (SD 35.42)	0.60^†^
MG	22.62 (SD 40.03)	0.54^†^
Pul	15.13 (SD 19.78)	0.18
VA	13.12 (SD 19.92)	0.08
VP	33.04 (SD 33.79)	0.01
*Others*		
Ins	8.35 (SD 10.28)	0.16
PVC	0.79 (SD 1.99)	0.31
RN	29.21 (SD 40.14)	0.03
SSAC	9.73 (SD 11.81)	0.06

^†^ *p* < 0.05. Abbreviations for VOIs: ACC, anterior cingulate cortex; DLPFC, dorsolateral prefrontal cortex; GPe, external segment of globus pallidus; GPi, internal segment of globus pallidus; IFG, inferior frontal gyrus; Ins, insula; LG, lateral geniculate body; MG, medial geniculate body; OFC, orbitofrontal cortex; Pul, pulvinar; Put, putamen; PVC, primary visual cortex; RN, red nucleus; SN, substantia nigra; SSAC, somatosensory association cortex; VA, ventral anterior nucleus; VP, ventral posterior nucleus; PD, Parkinson's disease; rCBF, regional cerebral blood flow; SD, standard deviation; VOI, volume of interest.

**Table 3:** rCBF changes in response to antiparkinsonian drugs in the SN, LG, and MG and their correlation with motor improvement: a subgroup analysis.

Group	Mean *rCBF change index*	*r*
*(a) SN*		
Total 30 patients	31.10 (SD 32.36)^†^	0.37^†^
Subgroups		
PD-ND	39.31 (SD 36.18)^†^	0.39
PD-D	21.72 (SD 24.14)^†^	0.16

*(b) LG*		
Total 30 patients	17.22 (SD 35.42)^†^	0.60^†^
Subgroups		
PD-ND	27.08 (SD 38.92)^†^	0.61^†^
PD-D	5.95 (SD 26.81)	0.47

*(c) MG*		
Total 30 patients	22.62 (SD 40.03)^†^	0.54^†^
Subgroups		
PD-ND	25.89 (SD 42.10)^†^	0.54^†^
PD-D	18.88 (SD 37.17)^†^	0.56^†^

^†^ *p* < 0.05. Abbreviations for VOIs: LG, lateral geniculate body; MG, medial geniculate body; SN, substantia nigra; PD-D, PD with dementia; PD-ND, PD without dementia; rCBF, regional cerebral blood flow; SD, standard deviation; VOI, volume of interest.

## Data Availability

The datasets analyzed in the present study are not publicly available, as they belong to the medical records of the hospital and the patients' privacy must be respected. Anonymized datasets are however available from the corresponding author upon reasonable request and with permission of an ethics review board.
